# DOF gene family expansion and diversification

**DOI:** 10.1590/1678-4685-GMB-2023-0109

**Published:** 2024-02-05

**Authors:** Edgar Luis Waschburger, João Pedro Carmo Filgueiras, Andreia Carina Turchetto-Zolet

**Affiliations:** 1 Universidade Federal do Rio Grande do Sul, Instituto de Biociências, Departamento de Genética, Programa de Pós-graduação em Genética e Biologia Molecular, Porto Alegre, RS, Brazil.

**Keywords:** Transcription factor, DOF, phylogeny, gene family expansion, adaptation

## Abstract

DOF (DNA binding with one finger) proteins are part of a plant-specific transcription factor (TF) gene family widely involved in plant development and stress responses. Many studies have uncovered their structural and functional characteristics in recent years, leading to a rising number of genome-wide identification study approaches, unveiling the DOF family expansion in angiosperm species. Nonetheless, these studies primarily concentrate on particular taxonomic groups. Identifying DOF TFs within less-represented groups is equally crucial, as it enhances our comprehension of their evolutionary history, contributions to plant phenotypic diversity, and role in adaptation. This review summarizes the main findings and progress of genome-wide identification and characterization studies of DOF TFs in Viridiplantae, exposing their roles as players in plant adaptation and a glimpse of their evolutionary history. We also present updated data on the identification and number of *DOF* genes in native and wild species. Altogether, these data, comprising a phylogenetic analysis of 2124 DOF homologs spanning 83 different species, will contribute to identifying new functional DOF groups, adding to our understanding of the mechanisms driving plant evolution and offering valuable insights into their potential applications.

## Introduction

Biotic and abiotic factors can limit plant growth, directly affecting the yield and quality of crop species. In native species, these factors can drive local adaptations, promoting species diversification and specialization. Abiotic stresses, such as extreme temperature, pH variation, high salinity, and drought, may become more common because of the current global climate crisis. In this manner, plants are inevitably confronted by different stress factors, threatening their growth and development. To deal with these stresses, plants undergo several metabolic and physiological changes by regulating specific stress-responsive genes. Ultimately, the development of climate-resilient genotypes can guarantee the survival of native species and the high productivity of crops.

Several transcription factors (TFs) have been described as involved in plant stress responses, some taking part in highly complex regulatory networks. Most of them are encoded by multigene families that experienced several rounds of gene duplication during land plant evolution (Riechmann et al., 2000; Wray et al., 2003). This expansion is suggested to be directly associated with organismal complexity, contributing to novel traits important for plant adaptation and agronomic-relevant traits (Lehti-Shiu et al., 2017). Likewise, the DOF (DNA binding with one finger) gene family exemplifies this expansion and diversification in angiosperms.

The DOF TFs are exclusive of Viridiplantae, having several roles in plant growth and development. DOF TFs have also been linked to biotic and abiotic responses. The DOF proteins generally comprise 200-400 amino acids and present two major regions in all their sequences: an N-terminal conserved DNA-binding domain (DOF domain) depicting a C2C2 type zinc finger motif and a C-terminal transcriptional regulatory region. The name DOF is due to the CX_2_CX_21_CX_2_C motif, which is predicted to form a single zinc finger domain protein (Yanagisawa, 2002) (Figure S1). The variability of several amino acid sequences at the transcriptional regulatory region of DOF proteins reflects their reported varied functions. In addition to its DNA-binding activity, the DOF domain presents a nuclear localization signal (Krebs et al., 2010). This characteristic, associated with the multiple roles that DOF proteins partake in, demonstrates the vital importance of these TFs in plants.

Since the first study of a *DOF* gene in 1995 (Yanagisawa, 1995), several studies have identified DOFs in various plant species (Gupta et al., 2015). Some demonstrated DOF protein functions and uncovered an increasing role diversity. Concomitantly, others performed *in silico* identification and characterization of *DOF* genes using the rising availability of plant genomes. These findings revealed that this gene family has expanded in angiosperms, and its diversity results from numerous duplication events (Moreno-Risueno et al., 2007). Although previous studies have explored aspects of their evolution, many questions remain about this gene family. Because most DOF studies focus on crop species, studying these genes in native species will contribute to knowledge of plant evolution and diversification. Furthermore, with the advancements in sequencing technologies and bioinformatics tools for manipulating data in the last ten years, conducting robust analyses is becoming more feasible. Here, we review the current knowledge of *DOF* genes based on a detailed examination of published articles to unveil the molecular evolution and diversification of this gene family and its potential role in plant adaptation. We summarize the main findings and progress of genome-wide identification and *in silico* characterization studies of *DOF* genes in Viridiplantae. We identified *DOF* genes in over 60 uncharacterized species using a bioinformatics approach. We performed an extensive phylogenetic analysis, including *DOF* genes of native and wild species, bringing to light this family’s complex evolutionary history.

## The current state of DOF TF research

**Figure 1 -:**
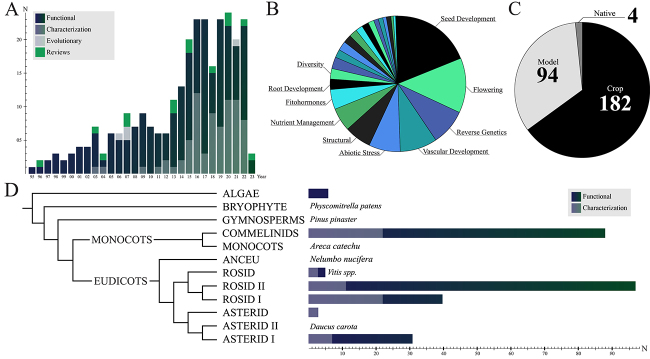
Data on DOF Literature. (A) Bar graph of articles published by year. Darker bars represent functional studies, gray bars identification/characterization studies, light gray evolutionary studies, and light green reviews. (B) Pie chart of the main topics of study discussed among articles. The ten most common topics are depicted. (C) Pie chart of main species studied. Black represents crop species, light gray model species, and dark gray native species. (D) Bar graph of the number of articles published by species in their botanical clades. The cladogram on the left depicts a representation of the phylogenetic relationships among different groups.

The first DOF TF family member was identified in 1995 (Yanagisawa, 1995), and 286 unique scientific research articles have been published since - as of February 2023 - half of which are dated to the last seven years (Figure 1A). This expansion in published articles is due mainly to increased genome-wide identification studies, while functional studies maintain similar numbers throughout the years. On the other hand, evolutionary studies are scarce and represent only four publications. As such, a lack of evolutionary studies suggests untapped biotechnological potential and a lack of guidance for characterization studies seeking orthologous gene classification and functional inference. The 196 functional studies were further classified into 25 research topics (Figure 1Band Table S1). Seed development and flowering are early identified pathways in DOF literature and the most common discussion topics, both earning their own summarized article reviews (Renau-Morata, et al., 2020a; Ruta et al., 2020). Their biotechnological potential makes the corresponding *DOF* genes compelling candidates for improving crop yield, with seed development focused on cereals and flowering in eudicots (Renau-Morata, *et al.*, 2020b). Not further behind, abiotic stresses, vascular development, and nutrient management represent the next agronomic traits of interest (Figure 1B).

Of the more than 70 reported species, *Arabidopsis thaliana* is the most studied, with 80 published articles, followed by *Oryza sativa* with 28 and *Zea mays* with 16. Although rice, maize, and many other economically important plants are considered model species in their genus and families, they were labeled crop species for this review. Moreover, nearly two-thirds of articles studied *DOF* genes in crop species, while 94 studied model species, including *Physcomitrella patens* (Sugiyama et al., 2012) and *Pinus pinaster* (Rueda-López et al., 2017) (Figure 1C). Only four articles were published focusing on *DOF* genes in native species *Chrysanthemum morifolium* (Song et al., 2016), *Tamarix hispida* (Yang et al., 2017), *Petunia inflata* (Yue et al., 2021), and *Eugenia uniflora* (Waschburger et al., 2022). A preference for studied species is also apparent when comparing the number of published articles considering the major botanical groups (Figure 1D). The Asterid clade refers to species with a common ancestral to Asterid I and II, and Rosid for Rosid I and II. Asterid II, Rosid, Ancient Eudicots, Monocots, Gymnosperms, and Bryophytes have only one species/genus studied, and alongside Algae, have a total of 16 published articles. The Rosid II clade is the most studied due to *A. thaliana*, closely followed by Commelinids, representing rice, maize, banana, pineapple, and other crops. Only one species was studied outside Commelinids, *Areca catechu* (Li et al., 2022). Rosid I, which includes Rosales and Fabales, is ahead of Asterid I, represented by Solanales, solely because of characterization studies.

Table S2 summarizes the number of DOF members across characterized *species* in genome-wide studies. This table comprises 72 articles encompassing 74 different species. It is important to note that in some studies, multiple species were characterized, and in some cases, more than one study characterized the same species. Among the genome-wide studies *Oryza sativa*, was included in five different articles, followed by *Gossypium hirsutum*, *Malus domestica*, *Musa acuminata*, *Populus trichocarpa*, *Solanum lycopersicum*, *Sorghum bicolor*, *Triticum aestivum*, and *Vitis vinifera*, which were included in three different studies each. Wheat shows the largest discrepancy in the number of predicted DOFs in its genome, ranging from 31 (Shaw et al., 2009) to 108 members (Fang et al., 2020). However, the authors posit that this number may be underestimated owing to ploidy and the preliminary state of the genome assembly. Gene duplication is an important mechanism for species evolution, which can occur individually or by whole-genome duplications (WGD). In plants and animals, WGD events are associated with adaptive radiations and evolutionary innovations (Pasquier et al., 2016). Gene duplications can arise from different mechanisms, which can leave marks in the genome, making it possible to estimate the process behind the duplication (Qiao et al., 2018). The authors have performed gene duplication analysis for 44 characterized species (Table S2). By compiling their results, it becomes clear that the primary source of new *DOF* paralogs is WGD/segmental duplications (37 species). Tandem duplication is the main diversity source of only three species; two species have similar segmental/tandem *DOF* paralogs; and the last two species present contrasting results in the literature, with one study proposing tandem duplications as predominant, while another proposing segmental duplication as predominant (Wang et al., 2018; Zou et al., 2019; Fang *et al.*, 2020; Liu et al., 2020). With these results, we can then correlate this increasing number of *DOF* paralogs found in Viridiplantae, from their single copy gene in algae to more than 100 members in some angiosperms, with the WGD events in plants, mainly in angiosperms (Vanneste et al., 2014), combined with the fact that transcription factors tend to be retained in the genome after duplication (Wang *et al.*, 2012).

These same duplicated genes have four possible fates: pseudogenization, neofunctionalization, subfunctionalization, and conservation, which can vary according to the acting selective pressure on paralogs (Magadum et al., 2013). A way to measure the selective pressure between paralogs is through non-synonymous (Ka) synonymous (Ks) substitution ratios. Among all genome-wide studies that sought to evaluate the selective force acting on the DOFs, most focused their analyses solely on pairs of paralogs, neglecting the comprehensive array of DOFs present within the species. Furthermore, these studies lack methodological information, and the results were little discussed/explored. The findings of the 37 species with Ka/Ks rate estimated (Table S2) reveal a predominant pattern of purifying selection among pairs of DOF paralogs. This result may be biased, as older duplications that underwent functional diversification may have accumulated enough mutations to the point that “its paralogous pair” could no longer be recovered. Therefore, there is a high chance that these works have evaluated only recent duplications at the beginning of their diversification process and/or old duplications under purifying selection. A contrasting result was found in *Jatropha curcas*, where 82% of the duplicated gene pairs analyzed presented Ka/Ks ratios greater than one, suggesting positive selection (Wang et al., 2018). Perhaps this result can be explained by the low similarity between the pairs of analyzed sequences, which ranged from 10.8% to 80% with a mean of 26.5%. Lastly, only four studies applied more robust methods of detecting selection forces. Based on Maximum Likelihood models (such as PAML and Datamonkey) or Bayesian models (such as the Selecton server) for ω estimates, three studies have shown that these sites are not within the DOF domain, and the other does not comment on whether sites under positive selection are or not present in the domain region.

## Phylogenetic analysis in taxonomic relevant species

**Figure 2 -:**
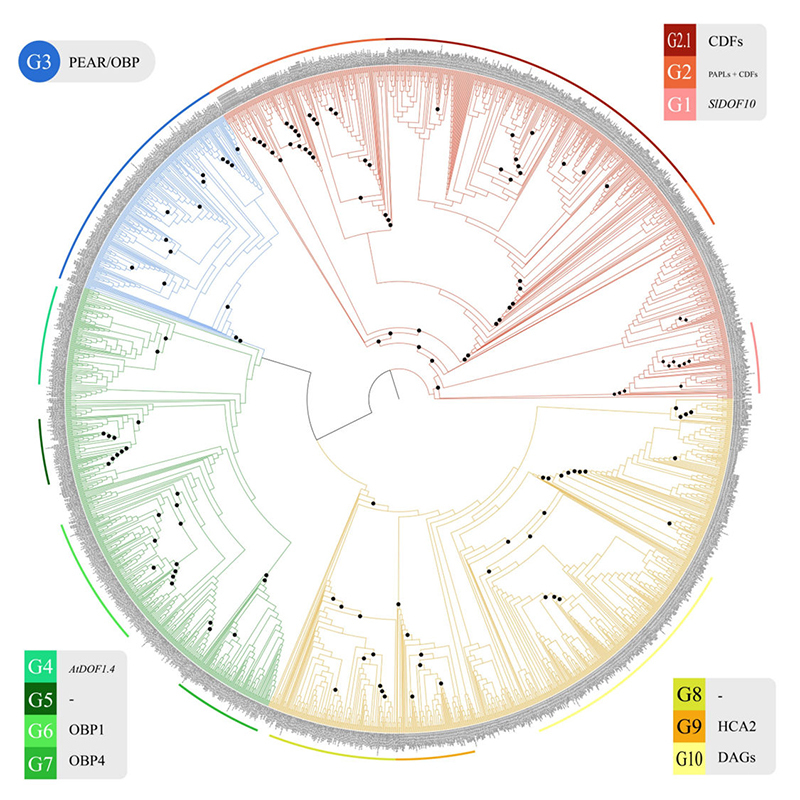
DOF TF Family Phylogeny. Maximum likelihood phylogeny of DOF proteins. Every different color represents a different functional group. Node circles represent a branch UFBootstrap support value over or equal to 85. Group names, codes, and respective species members are referred to in Table S4.

The first publications peering at DOF evolutionary relationships (Yanagisawa, 2002; Lijavetzky et al., 2003) were done in the early 2000s, when phylogenetic methodologies, data sources, and computational tools were much more limited. The study of the DOF gene family is challenging due to paralogous members per species and the short conserved domain. These studies became important stepstones in DOF characterization using *Arabidopsis thaliana* and *Oryza sativa* sequences. Since then, authors have refined their strategies, with Moreno-Risueno et al. (2007) adding more basal species (*Chlamydomonas reinhardtii*, *Physcomitrella patens*, *Selaginella moellerdorffii,* and *Pinus taeda*). Currently, proposed group classifications have little to no branch support, and as a consequence, groups tend to switch members, leading to low reproducibility between studies (Table S3). We have reconstructed a phylogeny of the DOF gene family to elucidate DOF TF family evolution, including 2124 DOF homologs from 82 species with filtered genes present in Material S1. Our DOF gene tree constructed with the ML method has recovered a total of four main clades (Figures 2 and S2) encompassing ten major groups with high branch support (UFBootstrap ≥ 85), representing three more groups than the highest number proposed in the literature (Moreno-Risueno *et al.,* 2007) (Figure 2). The phylogeny was rooted according to the presence of green algae sequences, as they represent the least complex organisms bearing DOF genes. Although some groups presented high UFBootstrap values, like those closely related to Group 10, they were not considered in this study since the genes displayed the highest sequence divergence from other *DOF* genes (Figure S2). Hence, a UFBootstrap support over 85 is likely due to long-branch attraction rather than proper sequence similarity. Furthermore, these same genes are the most likely to have risen from positive selection forces. Whether they are still under these same forces is beyond the methodological scope of this review. The full methodology can be found in Material S2.

Within our phylogeny, a total of 16 genes (three sequences from *Ceratodon purpureus*, four from *Physcomitrella patens*, five from *Aquilegia coerulea*, three from *Actinidia chinensis*, one from *Rhododendron delavayi*) had more than one DOF domain along their sequences. Thus, each domain was separated and treated individually (each domain was marked with a “_D#” at the end, “#” corresponding to the numerical order of the motif). Regardless, all domains originating from a common gene ended up grouped, implying the action of purifying selection forces to maintain these repeated domains instead of diversifying them. These genes containing more than one motif are a novelty among *DOF* genes and have not yet been reported nor functionally characterized. As such, we cannot be sure whether they are artifacts.

### The early stages of DOF evolution

Although early phylogenies struggle to group sequences with a complex evolutionary history and no clear relationships, such analyses can predict majorly conserved ones very well. Group 2, represented by PINEAPPLE (PAPL) and CYCLING DOF (CDF) genes, appears to be the most well-conserved and has been recovered by all previous phylogenies. The PAPL nomenclature was recently proposed by (Otero et al., 2022), for *COG1* (*PAPL1*) and *CDF4* (*PAPL2*) genes, based on their expression patterns. Shigyo et al. (2007) spotted a possible G2 division of PAPLs and CDFs, congruent with later studies identifying distinct expression patterns and biological functions (Fornara et al., 2009) for these same genes. Given these differences and the topology found in our phylogeny, it seems plausible that G2 presents two genetic lineages (PAPLs and CDFs). Although the phylogenetic analysis does not report a clear separation between the PAPL and the CDF, we can discuss and propose the CDFs as a specialized clade within G2, with both forming monophyletic groups. The most ancestral sequences of the G2 clade correspond to the characterized PAPL genes, in addition to grouping sequences belonging to algal and bryophyte species and containing few angiosperm sequences. On the other hand, the genes characterized as CDF appear within G2.1 as a more derived clade, with an absence of bryophytes and algae, in addition to a greater diversity of angiosperm sequences. The CDF subgroup (G2.1) probably originated between bryophytes and sporulating tracheophytes (ST), suggesting that the first *DOF* gene is closer to PAPL than CDF. Further studies comparing these two lineages will be important to support this hypothesis. In addition to the fact that *DOF* genes grew in numbers with the emergence of bryophytes, their diversification is not quite apparent yet. Although some bryophyte species have more than 20 DOF members, all their grouped sequences either belong to the ancestral PAPL lineage or are ungrouped, suggesting they diversified into more than one homologous group later in their evolutionary history. It would be very intriguing to see functional characterization studies of the bryophyte genes to understand how much they differ in functionality. Associated with the emergence of CDFs, Groups 4 and 5 possibly appeared during ST history and together would represent the oldest DOF groups after the G2 group. Unfortunately, both groups contain sequences from 1 out of the 3 ST species encompassed in the phylogeny (*Salvinia cucullata* with one sequence in Group 5 and *Selaginella moellendorffii* with four sequences in Group 6), thus making it hard to properly place the true origin of these groups, a limitation when dealing with taxonomic groups without much available genetic information or with early genome assembly phases. As such, it is very plausible that Group 4 only emerged during the surging of gymnosperms and Group 5 with the surging of eudicots (monocots were skipped because only one species out of the six present sequences within Group 5).

In conclusion, it is likely that the first *DOF* genes (Group 2) appeared in algae, experienced a rapid growth in members in bryophytes, and began diversifying into other homologous groups and lineages in ST species. This diversification resulted in the appearance of the CDF genes and possibly of Groups 4 and 5. Interestingly, since ST species do not produce buds or flowers, CDF genes likely originated to act in pathways other than those they were first discovered in (regulation of flowering).

### Expansion and diversification

**Figure 3 -:**
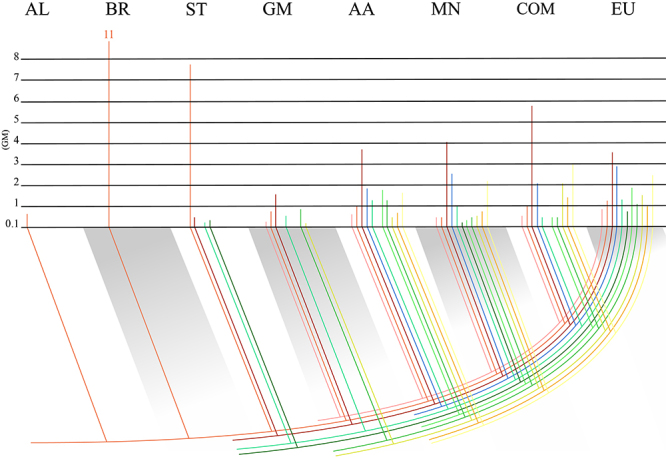
Homologous Groups Evolutionary Relationships. Colored lines illustrate the phylogenetic groups. The graph’s Y axis denotes the geometric mean of sequences in each homologous group. Values equal to 0.1 represent homologous groups with 0 sequences.

Though the DOF gene family had reached its usual size (20 - 40 members per diploid species) in bryophytes, a different type of expansion occurred following ST species. *DOF* genes began a steep increase in group numbers in gymnosperms while, at the same time, decreasing the number of genes present in the PAPL lineage. The geometric mean for the number of sequences in the PAPL lineage began at 11.44 in bryophytes, was reduced to 7.83 during STs, and went down to 0.75 in gymnosperms (Figure 3). During the same time, the CDF lineage emerged and achieved a mean of 1.55, which doubled to 3.68 in ancient angiosperm species. It is safe to say that Groups 1, 4, and 7 arose with the ancestor of gymnosperms, while Group 8 is less certain, thus duplicating the total number of groups. Similarly, Groups 3, 6, 8, 9, and 10 are present only in angiosperm species, which would represent another doubling in group numbers. This huge expansion is evidence of the high diversification *DOF* genes experienced, increasing to a twofold increase from STs. After angiosperms, groups seem to have stopped increasing in numbers, not considering the uncertainty of Group 5 that may have appeared later on. Considering our phylogeny holds 50 eudicot species from different genera, and no other group was found to be exclusive to clades such as Rosales or Asterids, it seems *DOF* genes have had their diversification decreased considerably. Since our research aims for a broader view and does not include as many monocot species, an interesting study with a complementary approach would focus on the DOF evolutionary history of monocots and search for possible exclusive groups, thus allowing a comparison of the diversification of these lineages. Another limitation to finding these groups and properly characterizing them in their evolutionary context is the preference for performing studies on crops over native species. The latter would provide information on unstudied genera and a glimpse over less anthropomorphized genomes, possibly under different environmental conditions and positive selection, which could lead to DOF diversification.

In conclusion, *DOF* genes have maintained overall numbers while highly diversifying their functions after STs. This diversification mainly happened during gymnosperms and angiosperms and ended up increasing group numbers by 2-folds. More recently, *DOF* genes seemingly lowered their diversification drive in angiosperms since no specific groups for Rosids or Asterids were detected. Albeit without complementary studies in native species or poorly represented angiosperm lineages, it is unclear whether this holds.

## Homologous groups and biological roles

Among the 11 different groups present, eight have functionally characterized genes. Unfortunately, Groups 4, 5, and 8 have no functionally characterized sequences. Table 1 presents a compilation of the main biological roles of the 11 different groups. The only characterized sequence of Group 1, *SlDOF10* (*Solyc02g090310*), is an orchestrator of vascular development during fruit ovary formation (Rojas-Gracia et al., 2019). Group 2 sequences have been shown to alter expression levels of *Phytochrome Interacting Factor* (PIF) proteins in *A. thaliana*. Not only are *AtCDF2* and *PIF4* temporally and spatially co-expressed, but the overexpression of *AtCDF3* from Group 2.2 led to higher amounts of *AtPIF1* mRNA while a loss of function mutant for *AtPAPL1* (*AtCOG1*) from Group 2.1 led to an increase in *AtPIF4* and *AtPIF5* mRNA levels (Fornara et al., 2009; Wei et al., 2017; Gao et al., 2022). Considering their common ancestry, the regulation and relationship with the bHLH PIF transcription factors are likely to be a shared characteristic. The main studied biological process of the PAPL lineage is the negative regulation of phytochrome A and B signaling pathways. Both PAPL1 and PAPL2 (*AtCDF4*) have been reported to regulate biosynthetic genes for GA, ABA, and BR hormones (Bueso et al., 2016; Wei *et al.*, 2017; Xu et al., 2020). Another function of PAPL2 is the regulation of floral organ abscission (Xu *et al.*, 2020). As for the CDF group, all *A. thaliana* proteins have been shown as direct repressors of *CONSTANS* (CO) and *FLOWERING LOCUS T* (FT) except for *AtCDF6*, thus acting redundantly in the repression of flowering. CDFs are also degraded by the circadian rhythm-responsive *GIGANTEA* (GI), *FLAVIN BINDING, KELCH REPEAT,* and *F-BOX 1* (FKF1) protein complex. A more in-depth review of the CDF subgroup, including their abiotic stress responses, was recently published (Renau-Morata et al., 2020a). Something worth noting about CDFs and glanced past in recent reviews is the capability of *AtCDF2* to interact with *DICER LIKE 1* (DCL1) and promote the transcription of miRNAs miR156 and miR172, further regulating the flowering process (Sun et al., 2015). This interaction could be shared among CDF proteins since, generally, their motifs are conserved, but no other has been reported to have such a capability.

**Table 1 -:** Overview of Homologous Groups Characteristics. The total number of sequences relates to our constructed phylogeny. The geometric means of the eudicot and monocot species in each group are displayed as well as *A. thaliana* and *O. sativa* sequences. The colors refer to the phylogenetic groups shown in Figure 2.

Group		Total Sequences	Eudicot GM	Monocot GM	A. thaliana	O. sativa	Biological Roles
1		68	0.85	0.53	0	0	Ovary development
2		210	1.25	0.95	2	2	Light signaling and hormonal responses
2.1		330	3.56	5.76	5	5	Repression of flowering, abiotic stresses
3		231	2.84	2.06	5	5	Vascular development, biotic stress and light signaling
4		95	1.28	0.45	1	0	Unkown
5		63	0.69	0.1	0	0	Unkown
6		143	1.8	0.45	3	1	Vascular development, auxin signal transduction, cell cycle progression and floral fate aquisition
7		86	1.07	0.49	1	2	ABA mediated repression of root growth
8		122	1.5	2.06	2	3	Unkown
9		76	0.95	1.41	1	1	Vascular development
10		224	2.41	2.99	5	2	Germination and light signaling

Many *DOF* genes have been reported as regulators of vascular development, especially root procambium formation. *AtDOF2.4* (PEAR1) and *AtDOF5.1* (PEAR2), from Group 3, are regulated by CK levels and promote procambium cell periclinal divisions (Miyashima et al., 2019), while also orchestrating leaf polarity (Kim et al., 2010). PEAR proteins are mobile TFs capable of traversing from the outer cambium to the inner cambium by symplastic trafficking, upon which they also promote the transcription of HD-ZIP III proteins. These same HD-ZIP III proteins, along with micro RNAs miR165 and miR166, repress PEAR protein activity in a negative loop manner (Miyashima *et al.*, 2019). Also, in Group 3, *AtDOF 1.1* (OBP2) has been shown to have phloem expression and act self-regulatory with PEAR proteins. Furthermore, OBP2 has altered expression levels in response to jasmonic acid treatment and herbivory while also regulating glucosinolates biosynthesis and positively regulating the number of vascular cell files (Skirycz et al., 2006; Qian et al., 2022). On the other hand, *AtDOF3.6* (OBP3) represses hypocotyl growth via *phyB* signaling pathway and cotyledon cell expansion via the blue-light sensitive protein *cry1* (Ward et al., 2005). In general, the sequences in Group 3 appear to have an ample relationship with vascular development while also connecting secondary pathways related to environmental sensors and stresses. The subsequent groups, 6 through 9, have had their sequences also reported to play major roles in vascular development processes. *AtDOF5.8* and *AtDOF3.4* (OBP1), both from Group 6, have been shown to be controlled by the auxin-orchestrator gene *MONOPTEROS* (MP) to regulate provascular cell divisions and cell expansion in root, shoot, and cotyledons (Skirycz *et al.*, 2008; Konishi and Yanagisawa, 2015). These genes have been recently reported to be expressed in primordia initiation and potentially link growth with floral fate acquisition (Larrieu et al., 2022). Interestingly, *AtDOF1.6* has had no functional study, nor has it been reported to act in these same pathways, even though it is present in this same group. *AtDOF5.4* (OBP4), from Group 7, acts as a mediator of ABA responses in repressing root hair growth by regulating cell cycle progression (Xu et al., 2016; Rymen et al., 2017), and *AtDOF5.6* (HCA2), from Group 9, promotes procambium cell divisions (Guo et al., 2009).

Much like Group 2, some genes from Group 10 are also included in signaling pathways involving PIF proteins. *AtDOF3.7* (DAG1) is positively regulated by *PIF1*, while *AtDOF2.5* (DAG2) is negatively regulated (Gabriele et al., 2009; Santopolo et al., 2015). Both proteins regulate DELLA proteins and biosynthetic genes for ABA and GA. While DAG1 appears to regulate germination negatively, DAG2 promotes it (Gualberti et al., 2002). DAG1 has also been found to induce hypocotyl growth via a complex hormonal network involving auxin, ethylene, and ABA (Lorrai et al., 2018). Apart from the DAG genes, *AtDOF4.6* (VDOF1) and *AtDOF1.8* (VDOF2) are related to leaf vein patterning and the repression of lignin biosynthesis and deposition (Ramachandran et al., 2020). Lastly, *AtDOF4.8* (ITD1) encodes a plasmodesma mobile protein without much functional information (Chen et al., 2013). Whether *ITD1* shares its trafficking motif with its orthologous sequences has not yet been elucidated.

## Conclusions, limitations, and perspectives

In conclusion, our exhaustive examination of the DOF literature, revisiting well-established topics and prospecting less-explored ones, allowed us to establish the foundation for future evolutionary studies and open new questions regarding this important gene family. We also investigated the evolutionary history of the DOF TF family and identified 10 majorly conserved and highly supported groups. DOF history is likely marked by constant duplication events followed by neofunctionalization ever since it got its first member. It has a significant family expansion with the emergence of angiosperm species around 150 million years ago. Some limitations of this study include the low number of basal species analyzed due to genome availability in online databases, bringing uncertainties about when certain major DOF groups arose. Furthermore, some species with available genomes are in the initial phases of assembly and annotation, more likely underestimating the number of *DOF* genes across species.

## Supplementary material

The following online material is available for this article:

Figure S1 - DOF domain representation.

Figure S2 - Unrooted phylogeny.

Table S1 - DOF TFs literature data.

Table S2 - DOF sequences diversification.

Table S3 - Comparison of DOF homologous groups among studies.

Table S4 - Phylogenetic groups sequence numbers.

Material S1 - Filtered sequences.

Material S2 - Materials and methods.
