# Supplementary material for: DOF gene family expansion and diversification
Source: Genet Mol Biol. 2024 Feb 5;46(3 Suppl 1):e20230109. doi: 10.1590/1678-4685-GMB-2023-0109 (PMC10842470; doi:10.1590/1678-4685-GMB-2023-0109)
Supplement: Table S1 - [file 1415-4757-GMB-46-03-s1-e20230109-s3.pdf]

Genetics and Molecular Biology
https://doi.org/10.1590/1678-4685-GMB-2023-0109

Supplementary Material to "DOF gene family expansion and diversification"

Table S1 - DOF TFs literature data.

Article Type	Author	Year	Species Foccused	Foccus	Main Topic	Main goal
Article	Yanagisawa	1995	*Z. mays*	Crop species	Structural	Domain analysis
Article	Paolis et al.	1996	*Nicotiana tabacum*	Crop species	Forward Genetics	Gene Elucidation
Review	Yanagisawa	1996			Structural	Motif of Zn-finger
Article	Vicente-Carbajosa et al.	1997	*Z. mays*	Crop species	Reverse Genetics	PBF characterization
Article	Yanagisawa	1997	*Z. mays*	Crop species	Structural	Physical interaction with DOFs
Article	Kisu et al.	1998	*Cucurbita maxima*	Crop species	Reverse Genetics	Gene Elucidation
Article	Mena et al.	1998	*H. vulgare*	Crop species	Seed Development	Pathway Elucidation
Article	Yanagisawa and Sheen	1998	*Z. mays*	Crop species	Reverse Genetics	Activity and regulation of Dofs
Article	Baumann et al.	1999	*N. tabacum*	Crop species	Root Development	Pathway Elucidation
Article	Yanagisawa and Schmidt	1999	*Z. mays*	Crop species	Reverse Genetics	Dof DNA-Binding site
Article	Kang and Singh	2000	*A. thaliana*	Model species	Plant Development	OBP family elucidation
Article	Papi et al.	2000	*A. thaliana*	Model species	Seed Development	Gene Elucidation
Article	Yanagisawa	2000	*Z. mays*	Crop species	Nutrient Management	C Metabolism
Article	Plesch et al.	2001	*S. tuberosum*	Crop species	Stoma Development	Pathway Elucidation
Article	Tepperman et al.	2001	*A. thaliana*	Model species	Light Perception	Define genes under phyA control
Article	Washio	2001	*O. sativa*	Crop species	Seed germination	Response to GA
Article	Yanagisawa	2001	*Z. mays*	Crop species	Structural	Mapping the functional domains of ZmDOF1
Article	Diaz et al.	2002	*H. vulgare*	Crop species	Seed Development	Pathway Elucidation
Article	Gualberti et al.	2002	*A. thaliana*	Model species	Seed Development	Pathway Elucidation
Article	Mena et al.	2002	*H. vulgare*	Crop species	Seed Development	Pathway Elucidation
Article	Papi et al.	2002	*A. thaliana*	Model species	Seed Development	Gene Elucidation
Article	Cavalar et al.	2003	*Z. mays*	Crop species	Structural	Motif Binding Ability
Review	Cooper et al.	2003	*O. sativa*	Crop species	Seed Development	Seed Coating & Abiotic Stress
Article	Isabel-LaMoneda	2003	*H. vulgare*	Crop species	Seed Development	Pathway Elucidation
Article	Kang et al.	2003	*A. thaliana*	Model species	Fitohormones	Pathway Elucidation
Article	Lijavetzki et al.	2003	*Oryza sativa*	Crop species	Characterization	O. sativa characterization
Article	Park et al.	2003	*A. thaliana*	Model species	Light Perception	Pathway Elucidation
Article	Washio	2003	*O. sativa*	Crop species	Seed Development	Response to GA

Article Type	Author	Year	Species Foccused	Foccus	Main Topic	Main goal
Article	Hwang	2004	*Z. mays*	Crop species	Seed Development	Pathway Elucidation
Article	Umemura et al.	2004	*C. maxima*	Crop species	Structural	Characteristics of the zinc finger of AOBP
Review	Yanagisawa	2004	*Arabidopsis thaliana*	Model species	Characterization	Overview of molecular characteristics and mechanisms
Article	Chen et al.	2005	*T. aestivum*	Crop species	Photosynthesis	Gene Elucidation
Article	Diaz et al.	2005	*H. vulgare*	Crop species	Seed Development	Pathway Elucidation
Article	Imaizumi et al.	2005	*A. thaliana*	Model species	Flowering	Pathway Elucidation
Article	Martínez et al.	2005	*H. vulgare*	Crop species	Seed Development	Pathway Elucidation
Article	Rogers et al.	2005	*A. thaliana*	Model species	Secondary Metabolism	
Article	Ward et al.	2005	*A. thaliana*	Model species	Light Perception	Pathway Elucidation
Article	Derory et al.	2006	*Quercus petraea*	Crop species	Flowering	Gene Identification
Article	Nakano et al.	2006	*A. thaliana*	Model species	Fitohormones	TFs cooperatively regulated by ethylene and jasmonate
Article	Ravel et al.	2006	*T. aestivum*	Crop species	Diversity	Genetic Diversity
Article	Skirycz et al.	2006	*A. thaliana*	Model species	Reverse Genetics	AtDof1.1 (OBP2) characterization
Article	Yamamoto et al.	2006	*O. sativa*	Crop species	Seed Development	Pathway analysis
Article	Yang et al.	2006	*Review*	Model species	Evolution	Evolutionary paths of paralogs
Article	Chawade et al.	2007	*A. thaliana*	Model species	Abiotic Stress	Cold Stress
Article	Dong et al.	2007	*T. aestivum*	Crop species	Seed Development	Pathway Elucidation
Article	Konishi and Yanagisawa	2007	*A. thaliana*	Model species	Vascular Development	Gene Elucidation
Review	Moreno-Risueno (A)	2007	*Review*	Model species	Evolution	Evolution elucidation
Article	Moreno-Risueno (B)	2007	*H. vulgare*	Crop species	Seed Development	Pathway Elucidation
Article	Nakamichi et al.	2007	*A. thaliana*	Model species	Flowering	Pathway Elucidation
Review	Shigyo et al.	2007	*Review*	Model species	Evolution	Evolution elucidation
Article	Skirycz et al.	2007	*A. thaliana*	Model species	Secondary Metabolism	Function of AtDOF4;2 in the phenylpropanoid metabolism
Article	Wang et al.	2007	*G. max*	Crop species	Seed development	Lipid pathways
Article	Ito et al.	2008	*A. thaliana*	Model species	Flowering	Pathway Elucidation
Article	Li et al.	2008	*O. sativa*	Crop species	Reverse Genetics	Gene Elucidation
Article	Marzábal et al.	2008	*Z. mays*	Crop species	Seed Development	Gene Elucidation
Article	Rueda-López et al.	2008	*Pinus pinaster*	Model species	Reverse Genetics	PpDof5 identification and characterization
Article	Skirycz et al.	2008	*A. thaliana*	Model species	Cell Cycle	DOF (OBP1) in the Cell Cycle regulation
Article	Wu et al.	2008	*A. thaliana*	Model species	Flowering	Pathway/regulation of Photoperiodic flowering
Article	Zou et al.	2008	*H. vulgare*	Crop species	Seed Development	TF interactions in Aleurone Cells
Article	Fornara et al.	2009	*A. thaliana*	Model species	Flowering	Pathway Elucidation
Article	Guo et al.	2009	*A. thaliana*	Model species	Vascular Development	Gene Elucidation

Article Type	Author	Year	Species Foccused	Foccus	Main Topic	Main goal
Article	Iwamoto et al.	2009	*O. sativa*	Crop species	Flowering	Pathway Elucidation
Article	Kumar et al.	2009	*T. aestivum*	Crop species	Nutrient Management	N Metabolism
Article	Li et al.	2009	*O. sativa*	Crop species	Reverse Genetics	Molecular characterization
Article	Shaw et al.	2009	*Triticum aestivum*	Crop species	Characterization	T. aestivum characterization
Article	Tanaka et al.	2009	*I. batatas*	Crop species	Nutrient Metabolism	Starch storage in roots
Article	Tsujimoto et al.	2009	*A. thaliana*	Model species	Nutrient Management	C/N Metabolism
Article	Xin et al.	2009	*Populus tomentosa*	Crop species	Reverse Genetics	Characterization of PtDof1
Article	Gabriele et al.	2010	*A. thaliana*	Model species	Seed germination	Effect of DAG1 on germination
Article	Gardiner et al.	2010	*A. thaliana*	Model species	Vascular Development	Gene Elucidation
Article	Kawakatsu et al.	2010	*O. sativa*	Crop Species	Seed Development	Pathway Elucidation
Article	Kim et al.	2010	*A. thaliana*	Model species	Fitohormones	Effect of Dof5.1 leaf polarity
Article	Krebs et al.	2010	*A. thaliana*	Model species	Structural	Nuclear Localization
Article	Wei et al.	2010	*A. thaliana*	Model species	Organ Abscission	Gene Elucidation
Article	Yang et al.	2010	*J. curcas*	Crop species	Flowering	Gene Elucidation
Article	Cominelli et al.	2011	*A. thaliana*	Model species	Structural	Promoter Dissection
Article	Gaur et al.	2011	*O. sativa*	Crop species	Seed Development	Transcriptional profiling of DOFs in seed development
Article	Gupta et al.	2011	*E. coracana*	Crop Species	Diversity	Gene Elucidation
Article	Kushwaha et al.	2011	*Sorghum bicolor*	Crop species	Characterization	S. bicolor characterization
Article	Rizza et al.	2011	*A. thaliana*	Model species	Seed Development	Effects of ELIP1 and 2 on Seed Germination
Article	Yang et al.	2011	*J. curcas*	Crop species	Flowering	Gene Elucidation
Article	Barajas-Lopez et al.	2012	*Pisum sativum*	Crop species	Nutrient Management	Effect of Sugar on Photosynthesis
Article	Chen et al.	2012	*Z. mays*	Crop species	Reproduction	Pollen development
Article	Gupta et al.	2012	*E. coracana*	Crop Species	Diversity	Gene Elucidation
Article	Hernando-Amado et al.	2012	*Brachypodium distachyon*	Crop species	Characterization	B. distachyon characterization
Article	Rueda-Romero et al.	2012	*A. thaliana*	Model species	Seed germination	DOF6 in the seed germination
Article	Sugiyama et al.	2012	*Physcomitrella patens*	Model species	Characterization	P. patens characterization and functionally analysis
Article	Ahmad et al.	2013	*A. thaliana*	Model species	Vascular Development	Gene Elucidation
Article	Cai et al.	2013	*Solanum lycopersicum*	Crop species	Characterization	S. lycopersicum characterization
Article	Chen et al.	2013	*A. thaliana*	Model species	Structural	Cell Trafficking
Article	Kloosterman et al.	2013	*Solanum tuberosum*	Crop species	Root Development	Tuberization
Article	Kushwaha et al.	2013	*S. bicolor*	Crop Species	Structural	Tertiary Structure
Review	Le Hir and Bellini	2013	*A. thaliana*	Model species	Vascular Development	Vascular Development
Article	Negi et al.	2013	*A. thaliana*	Model species	Stoma Development	SCAP1 on Guard-Cells development

Article Type	Author	Year	Species Foccused	Foccus	Main Topic	Main goal
Review	Noguero et al.	2013	*Review*	Crop species	Review	Review
Article	Takano et al.	2013	*N. tabacum*	Crop species	Biotic Stress	Pathogen Defense
Article	Wang et al.	2013	*A. thaliana*	Model species	Nutrient Management	N Metabolism
Article	Zou et al.	2013	*A. thaliana*	Model species	Reverse Genetics	Gene Elucidation
Article	Boccaccini et al. (A)	2014	*A. thaliana*	Model species	Seed Development	Functional relationship between DAG1, RGA and GAI in seed germination
Article	Boccaccini et al. (B)	2014	*A. thaliana*	Model species	Seed Development	GA3ox1 gene regulation
Article	Corrales et al.	2014	*S. lycopersicum*	Crop species	Flowering	Abiotic Stress Relationship
Article	Gupta et al.	2014	*E. coracana*	Crop species	Reverse Genetics	Gene Elucidation
Article	Gupta et al.	2014	*E. coracana*	Crop Species	Nutrient Management	Circadian Rythm/Nitrogen
Article	Ibánez-Salazar et al.	2014	*C. reinhardtii*	Model species	Lipid Metabolism	Gene Elucidation
Article	Jin et al.	2014	*Ricinus communis*	Crop Species	Characterization	R. communis Characterization
Article	Kumar et al.	2014	*E. coracana*	Crop Species	Reverse Genetics	Gene Elucidation
Article	Liang et al.	2014	*A. thaliana*	Model species	Global Study	miRNA
Article	Malviya et al.	2014	*Cajanus cajan*	Crop species	Characterization	C. cajan characterization
Article	Mehrotra et al.	2014	*A. thaliana*	Model Species	Structural	Genome wide identification of DOF-DNA binding motif
Article	Park et al.	2014	*O. sativa*	Crop species	Biotic Stress	Leaf Wounding
Article	Yu et al.	2014	*G. max*	Crop species	Fitohormones	Gene Elucidation
Article	Chen and Cao	2015	*Z. mays*	Crop species	Characterization	Z. mays Characterization and DOF evolution
Article	Ferreira et al.	2015	*O. sativa*	Crop Species	Nutrient Management	N Metabolism
Article	Fornara et al.	2015	*A. thaliana*	Model species	Flowering	Pathway Elucidation
Review	Gupta et al.	2015	*Review*	Crop species	Structural	Review
Article	Hamzeh-Mivehroud et al.	2015	*A. thaliana*	Model species	Structural	3D structure of DOF
Article	He et al.	2015	*A. thaliana*	Model species	Abiotic Stress	Salt, drought and ABA
Article	Huang et al.	2015	*Daucus carota*	Crop species	Characterization	D. carota characterization
Article	Konishi et al.	2015	*A. thaliana*	Model species	Vascular Development	Gene Elucidation
Article	Kushwaha et al.	2015	*Cereals*	Crop Species	Diversity	Genetic Diversity
Article	Lucas-Reina et al.	2015	*C. reinhardtii*	Model species	Flowering	Photoperiod
Article	Ma et al.	2015	*Brassica rapa*	Model species	Characterization	B. rapa characterization
Article	Noguero et al.	2015	*M. truncatula*	Crop species	Seed Development	Gene Elucidation
Article	Santopolo et al.	2015	*A. thaliana*	Model species	Seed Development	Gene Elucidation
Article	Sasaki et al.	2015	*N. tabacum*	Crop species	Biotic Stress	Pathogen Defense
Review	Seaton et al.	2015	*A. thaliana*	Model species	Flowering	Pathway Elucidation
Article	Shu et al.	2015	*Medicago truncatula*	Model species	Characterization	M. truncatula characterization
Article	Sun et al.	2015	*A. thaliana*	Model species	Flowering	miRNA

Article Type	Author	Year	Species Foccused	Foccus	Main Topic	Main goal
Article	Venkatesh and Park	2015	*Solanum tuberosum*	Crop species	Characterization	S. tuberosum characterization
Article	Wu et al.	2015	*O. sativa*	Crop species	Fitohormones	Brassinosteroids
Article	Zhang et al.	2015	*O. sativa*	Crop species	Photosynthesis	Gene Elucidation
Article	Boccaccini et al.	2016	*A. thaliana*	Model species	Seed Development	Gene Elucidation
Article	Bueso et al.	2016	*A. thaliana*	Model species	Seed Development	Seed Coating
Article	Cai et al.	2016	*S. lycopersicum*	Crop species	Abiotic Stress	Salt Stress
Article	Castorina et al.	2016	*A. thaliana*	Model species	Stoma Development	Gene Elucidation
Article	Chen et al.	2016	*Prunus persica*	Crop species	Characterization	P. persica characterization
Article	da Silva et al.	2016	*Vitis vinifera*	Crop species	Characterization	V. vitifera characterization
Article	Dong et al.	2016	*Musa acuminata*	Crop species	Characterization	M. acuminata characterization
Article	Feng et al.	2016	*Musa acuminata*	Crop species	Characterization	M. acuminata characterization
Article	Gupta et al.	2016	*Sorghum bicolor*	Crop species	Characterization	Gene Elucidation
Article	Iwamoto et al.	2016	*O. sativa*	Crop species	Seed Development	Pathway Elucidation
Article	Kang et al.	2016	*Capsicum annuum*	Crop species	Characterization	C. annuum characterization
Article	Li et al.	2016	*Camellia sinensis*	Crop species	Characterization	C. sinensis characterization
Article	Ma et al.	2016	*T. aestivum*	Crop species	Abiotic Stress	Salt stress
Article	Nasim et al.	2016	*Cicer arietinum*	Crop Species	Characterization	C. arietinum Characterization
Article	Ridge et al.	2016	*P. sativum*	Crop species	Flowering	Gene Elucidation
Article	Song et al.	2016	*Chrysantemum morifolium*	Native species	Characterization	C. morifolium characterization
Article	Wang et al.	2016	*A. thaliana*	Model species	Organ Abscission	Gene Elucidation
Article	Wang et al.	2016	*Phyllostachys heterocycla*	Crop species	Characterization	P. heterocycla characterization
Article	Wen et al.	2016	*Cucumis sativus*	Crop species	Characterization	C. sativus characterization
Article	Wu et al.	2016	*Capsicum annuum*	Crop species	Characterization	C. annuum characterization
Article	Wu et al.	2016	*C. sinensis*	Crop species	Fruit Development	Fruit Ripening
Article	Xu et al.	2016	*A. thaliana*	Model species	Cell Cycle	Gene Elucidation
Article	Xu et al.	2016	*B. napus*	Crop species	Flowering	Gene Elucidation
Article	Chang et al.	2017	*S. tuberosum*	Crop species	Reproduction	Pollen Development
Article	Corrales et al.	2017	*A. thaliana*	Model species	Flowering	Gene Elucidation
Article	Deeba et al.	2017	*O. sativa*	Crop Species	Abiotic Stress	Gene Elucidation
Article	Ge et al.	2017	*Phyllostachys edulis*	Crop Species	Flowering	Gene Elucidation
Article	Goralogia et al.	2017	*A. thaliana*	Model species	Flowering	Pathway Elucidation
Article	Henriques et al.	2017	*A. thaliana*	Model species	Flowering	Pathway Elucidation
Article	Ito et al.	2017	*Phaseolus vulgaris*	Crop species	Characterization	P. vulgaris characterization
Article	Molina-Hidalgo	2017	*Fragaria spp.*	Crop species	Secondary Metabolism	Gene Elucidation

Article Type	Author	Year	Species Foccused	Foccus	Main Topic	Main goal
Article	Ohtani et al.	2017	*A. thaliana*	Model Species	Vascular Development	Secondary Growth
Article	Peng et al.	2017	*Z. mays*	Crop species	Reproduction	Gene Elucidation
Article	Qi et al.	2017	*Z. mays*	Crop species	Seed Development	Starch Synthesis
Article	Ravindran et al.	2017	*A. thaliana*	Model species	Seed Development	Pathway Elucidation
Article	Renau-Morata et al.	2017	*S. lycopersicum*	Crop species	Abiotic Stress	Salt Stress
Article	Rouhian et al.	2017	*H. vulgare*	Crop species	Population Study	Genetic Diversity
Article	Rueda-López et al.	2017	*Poplar spp.*	Crop species	Nutrient Management	C/N Metabolism
Article	Rymen et al.	2017	*A. thaliana*	Model species	Root Development	Gene Elucidation
Article	Su et al.	2017	*G. hirsutum*	Crop species	Abiotic Stress	Salt Stress
Article	Wang et al.	2017	*Populus trichocarpa*	Model species	Characterization	P. trichocarpa characterization
Article	Wei et al.	2017	*A. thaliana*	Model species	Fitohormones	Pathway Elucidation
Article	Wu et al.	2017	*O. sativa*	Crop species	Flowering	Gene Elucidation
Article	Wu et al.	2017	*O. sativa*	Crop species	Nutrient Management	N Metabolism
Article	Yang et al.	2017	*Tamarix hispida*	Native species	Abiotic Stress	Salt Stress
Article	Zhang et al.	2017	*Setaria italica*	Model species	Characterization	S. italica characterization
Article	Azam et al.	2018	*Ananas comosus*	Crop species	Characterization	A. comosus characterization
Article	Cheng et al.	2018	*Phyllostachys edulis*	Crop species	Characterization	P. edulis characterization
Article	Gupta et al.	2018	*Eleusine coracana*	Crop species	Characterization	E. coracana characterization
Article	Krahmer et al.	2018	*A. thaliana*	Model species	Flowering	Circadian Rythm
Article	Li et al.	2018	*Gossypium hirsutum*	Crop Species	Characterization	G. hirsutum Characterization
Article	Lorrai et al	2018	*A. thaliana*	Model species	Plant Development	Hypocotyl growth
Article	Pandey et al.	2018	*T. aestivum*	Crop species	Structural	Mutation effect on DOF domain
Review	Pérez-Alonso et al.	2018			Global Study	Pipeline for combination of transcriptomics and metabolomics, with CDF3 in example
Article	Salas-Montantes et al.	2018	*C. reinhardtii*	Model species	Lipid Metabolism	Biofuel production
Article	Sani et al.	2018	*A. thaliana*	Model species	Structural	Biophysical properties
Article	Wang et al.	2018	*Jatropha curcas*	Crop species	Characterization	J. curcas characterization
Article	Wei et al.	2018	*Solanum melongena*	Crop species	Characterization	S. melongena characterization
Article	Xu et al.	2018	*Boehmeria nivea*	Crop species	Characterization	B. nivea characterization
Article	Yang et al.	2018	*Malus domestica*	Crop species	Characterization	M. domestica characterization
Article	Yang et al.	2018	*J. regia*	Crop species	Abiotic Stress	Heat Stress
Article	Zhang et al.	2018	*Malus domestica*	Crop species	Characterization	M. domestica characterization
Article	Guaberto et al.	2019	*Citrus sinensis*	Crop species	Characterization	C. sinensis characterization
Article	Guo et al.	2019	*Vigna radiata*	Crop species	Vascular Development	Petal Vasculature
Article	Hong et al.	2019	*A. comosus*	Crop species	Physiological Disorder	DOFs involved with Internal Browning (IB)
Article	Iwamoto et al.	2019	*O. sativa*	Crop species	Seed Development	Pathway Elucidation

Article Type	Author	Year	Species Foccused	Foccus	Main Topic	Main goal
Article	Jia et al.	2019	*C. reinhardtii*	Model species	Lipid Metabolism	Gene Elucidation
Article	Khaksar et al.	2019	*D. zibethinus*	Crop species	Characterization	D. zibethinus characterization
Article	Kondhare et al.	2019	*S. tuberosum*	Crop species	Root Development	Tuberization
Article	Liu et al.	2019	*Pyrus bretschneideri*	Crop species	Characterization	P. bretschneideri characterization
Article	Liu et al.	2019	*P. edulis*	Crop species	Flowering	Gene Elucidation
Article	Miyashima et al.	2019	*A. thaliana*	Model species	Vascular Development	Pathway Elucidation
Article	Qin et al.	2019	*O. sativa*	Crop species	Root Development	Ethylene Pathway
Article	Rojas-Gracia et al.	2019	*S. lycopersicum*	Crop species	Vascular Development	Gene Elucidation
Article	Shim et al.	2019	*O. sativa*	Crop species	Fitohormones	Jasmonate Leaf Senescence
Article	Smet et al.	2019	*A. thaliana*	Model species	Vascular Development	Pathway Elucidation
Article	Tokunaga et al.	2019	*Chlorella vulgaris*	Model species	Reverse Genetics	DOF overexpression effects
Article	Wang et al.	2019	*11 species*	Crop species	Characterization	Characterization
Article	Wu et al.	2019	*Z. mays*	Crop species	Seed Development	Starch Synthesis
Article	Wu et al.	2019	*Z. mays*	Crop species	Seed Development	Starch synthesis pathway
Article	Yu et al.	2019	*V. vinifera*	Crop species	Biotic Stress	Fungi infection
Article	Zhuo et al.	2019	*A. thaliana*	Model species	Fitohormones	Jasmonate Pathway
Article	Zou and Yang	2019	*Hevea brasiliensis*	Crop species	Characterization	H. brasiliensis characterization
Article	Zou and Zhang	2019	*Jatropha curcas, Ricinus communis*	Crop species	Characterization	Genome-wide identification of Dof family genes in J. curcas and R. communis
Article	Zou et al.	2019	*Manihot esculenta*	Crop species	Characterization	M. esculenta characterization
Article	Cai et al.	2020	*Saccharum spontaneum*	Crop species	Characterization	S. spontaneum characterization
Article	Cao et al.	2020	*Medicago sativa*	Crop species	Characterization	M. sativa charaterization
Article	Chattha et al.	2020	*Gossypium spp.*	Crop species	Characterization	Gossypium family characterization
Article	Chen et al.	2020	*M. domestica*	Crop species	Abiotic Stress	Drought Stress
Article	Domínguez-Figueroa et al.	2020	*A. thaliana*	Crop species	Nutrient Management	Gene Elucidation
Article	Fang et al.	2020	*Triticum aestivum*	Crop species	Characterization	T. aestivum characterization
Article	Gonzales et al.	2020	*S. tuberosum*	Crop species	Abiotic Stress	Drought
Article	Huang et al.	2020	*O. sativa*	Crop species	Seed Development	Heading Date in japonica and indica
Article	Iwamoto et al.	2020	*O. sativa*	Crop species	Seed Development	Pathway Elucidation
Article	Li et al.	2020	*Gossypium spp.*	Crop species	Characterization	Gossypium family characterization
Article	Liu et al.	2020	*Triticum aestivum*	Crop species	Characterization	T. aestivum characterization
Article	Martín et al	2020	*A. thaliana*	Model species	Flowering	Pathway Elucidation
Article	Mulat et al.	2020	*Eragrostis tef*	Crop species	Characterization	E. tef Characterization
Article	Nilsen et al.	2020	*T. aestivum*	Crop species	Vascular Development	Gene Elucidation
Article	Pandey et al.	2020	*Triticum aestivum*	Crop species	Characterization	T. aestivum Chr2 Characterization
Article	Qi et al.	2020	*M. domestica*	Crop species	Flowering	Gene Elucidation

Article Type	Author	Year	Species Foccused	Foccus	Main Topic	Main goal
Article	Ramachandram et al.	2020	*A. thaliana*	Model species	Vascular Development	Pathway Elucidation
Article	Renau-Morata et al.	2020	*S. lycopersicum*	Crop species	Fruit Development	Gene Elucidation
Review	Renau-Morata et al.	2020			CDF Review	Focus on CDF in the control of abiotic stress responses
Review	Ruta et al.	2020			Seed Development	DOFs involved in seed development
Article	Shangguan et al.	2020	*Vitis vinifera*	Crop species	Characterization	V. vinifera characterization
Article	Xu et al.	2020	*A. thaliana*	Model species	Organ Abscission	Gene Elucidation
Article	Yu et al.	2020	*Camellia sinensis*	Crop species	Characterization	C. sinensis characterization
Article	Zhou et al.	2020	*Citrullus lanatus*	Crop species	Characterization	C. lanatus Characterization
Article	Guo et al.	2021	*M. truncatula*	Crop species	Abiotic Stress	Gene Elucidation
Article	Hamdi et al.	2021	*Beta vulgaris*	Crop species	Characterization	B. vulgaris Characterization
Article	Iwamoto et al.	2021	*O. sativa*	Crop species	Seed Development	Pathway Elucidation
Article	Jain et al.	2021	*C. cajan*	Crop species	Abiotic Stress	Salinity stress
Article	Kandjani et al.	2021	*A. thaliana*	Model species	Diversity	Genetic Diversity
Article	Kandjani et al.	2021	*A. thaliana*	Model species	Reverse Genetics	Mutations
Article	Khan et al.	2021	*Oryza sativa*	Crop species	Characterization	O. sativa characterization
Article	Khan et al.	2021	*Juglans regia*	Crop Species	Characterization	J. regia Characterization
Article	Li et al.	2021	*V. yeshanensis*	Crop Species	Abiotic Stress	Gene Elucidation
Article	Liu et al.	2021	*O. sativa*	Crop species	Characterization	O. sativa characterization
Article	Lohani et al.	2021	*Brassica napus*	Crop species	Characterization	B. napus characterization
Article	Nan et al.	2021	*Rosa chinensis*	Crop species	Characterization	R. chinensis characterization
Article	Sun et al.	2021	*Betula platyphylla*	Crop species	Characterization	White birch characterization
Article	Wang et al.	2021	*Cleistogenes songorica*	Crop species	Characterization	C. songorica characterization
Article	Wang et al.	2021	*Vitis vinifera*	Crop species	Characterization	V. vinifera characterization
Article	Wang et al.	2021	*B. rapa*	Crop species	Organ Abscission	Leaf Senescence
Article	Xu et al.	2021	*S. lycopersicum*	Crop species	Flowering	Pathway Elucidation
Article	Yu et al.	2021	*Spinaceae oleracea*	Crop species	Characterization	S. oleraceae characterization
Article	Yu et al.	2021	*O. sativa, S. bicolor*	Crop species	Evolution	Structural Elucidation
Article	Yu et al.	2021	*Spinacia oleraceae*	Crop species	Characterization	S. oleraceae characterization
Article	Yue et al.	2021	*Petunia inflata*	Native species	Characterization	P. inflata characterization
Article	Arenas et al.	2022	*T. turgidum*	Crop species	Seed Development	Grain filling during short-term heat stress
Article	Blair et al.	2022	*A. thaliana*	Model species	Reverse Genetics	Gene Elucidation
Article	Cao et al.	2022	*Nelumbo nucifera*	Crop species	Characterization	N. nucifera characterization
Article	Gandass and Salvi	2022	*O. sativa*	Crop species	Reverse Genetics	Thermotolerance
Article	Gao et al.	2022	*A. thaliana*	Model species	Reverse Genetics	Hypocotyl growth
Article	Jia et al.	2022	*C. reinhardtii*	Model species	Lipid Metabolism	Pathway Elucidation

Article Type	Author	Year	Species Foccused	Foccus	Main Topic	Main goal
Article	Larrieu et al.	2022	*A. thaliana*	Model species	Flowering	Pathway Elucidation
Article	Li et al.	2022	*Vaccinium corymbosum*	Crop species	Characterization	Blueberry characterization
Article	Li et al.	2022	*Areca catechu*	Crop species	Characterization	A. catechu characterization
Article	Liu et al.	2022	*A. thaliana*	Model species	Vascular Development	Pathway Elucidation
Article	Luengwilai et al.	2022	*S. lycopersicum*	Crop species	Nutrient Management	Carbon and Nitrogen Use
Article	Luo et al.	2022	*Camelina sativa*	Crop species	Characterization	C. sativa characterization
Article	Moriwaki et al.	2022	*A. thaliana*	Model species	Stoma Development	Gene Elucidation
Article	Otero et al.	2022	*A. thaliana*	Model species	Vascular Development	Pathway Elucidation
Article	Qian et al.	2022	*A. thaliana*	Model species	Vascular Development	Pathway Elucidation
Article	Tabassum et al.	2022	*Oryza spp.*	Crop species	Characterization	Oryza wide characterization
Article	Wang et al.	2022	*Populus simonii x Populus nigra*	Crop species	Characterization	Populus characterization
Review	Wang et al.	2022			Review	Fitohormonal and stress review
Article	Waschburger et al.	2022	*E. uniflora*	Native species	Characterization	E. uniflora characterization
Article	Xiao et al.	2022	*Sorghum bicolor*	Crop species	Seed Development	Starch Biosynthesis
Article	Yang et al.	2022	*C. japonica*	Crop species	Characterization	C. japonica characterization
Article	Zhai et al.	2022	*Prunus avium*	Crop species	Fitohormones	Pathway Elucidation
Article	Zhang et al.	2022	*A. thaliana*	Model species	Vascular Development	Wound healing
Article	Ma et al.	2023	*Litchi chinensis*	Crop species	Organ Abscission	Gene Elucidation
Article	Wei et al.	2023	*G. max*	Crop species	Abiotic Stress	Pathway Elucidation
Review	Zou and Sun	2023			Review	Functions
