# Supplementary material for: DOF gene family expansion and diversification
Source: Genet Mol Biol. 2024 Feb 5;46(3 Suppl 1):e20230109. doi: 10.1590/1678-4685-GMB-2023-0109 (PMC10842470; doi:10.1590/1678-4685-GMB-2023-0109)
Supplement: Table S2 - [file 1415-4757-GMB-46-03-s1-e20230109-s4.pdf]

Genetics and Molecular Biology
https://doi.org/10.1590/1678-4685-GMB-2023-0109

Supplementary Material to "DOF gene family expansion and diversification"

Table S2 - DOF sequences diversification.

Species	DOFs members	Main source of diversification	Selection analysis	Reference	Predominant selective force
Actinidia chinensis	25	---	No	Shangguan et al. 2020	---
Aegilops tauschii	10	---	No	Wang et al. 2019	---
Ananas comosus	26	---	No	Azam et al. 2018	---
Ananas comosus	26	---	No	Shangguan et al. 2020	---
Arabidopsis thaliana	36	---	No	Wang et al. 2019	---
Arabidopsis thaliana	36	Segmental and Tandem duplication	Ka/Ks ratios	Shangguan et al. 2020	Purifying selection
Areca catechu	36	---	No	Li et al. 2022	---
Beta vulgaris	22	Segmental duplication	No	Hamdi et al. 2021	---
Betula platyphylla	26	inter-chromosomal duplication	Ka/Ks ratios	Sun et al. 2021	Purifying selection (all Less than 0.256)
Boehmeria nivea	19*	---	No	Xu et al. 2018	---
Brachypodium distachyon	29	---	No	Wang et al. 2019	---
Brachypodium distachyon	27	---	No	Hernando-Amado et al. 2012	---
Brassica napus	117	Segmental duplication	Ka/Ks ratios	Lohani et al. 2021	Purifying selection
Brassica rapa	76	Probably segmental duplication	No	Ma et al. 2015	---
Cajanus cajan	38	Tandem duplication	Ka/Ks ratios	Malviya et al. 2014	Ka/Ks <1 (5 pairs); Ka/Ks = 1 (5 pairs); Ka/Ks >1 (4 pairs)
Camelina sativa	103	Segmental duplication	Ka/Ks ratios	Luo et al. 2022	Purifying selection (all Less than 0.8, only one with 1.061

Species	DOFs members	Main source of diversification	Selection analysis	Reference	Predominant selective force
Camellia sinensis	29*	---	No	Li et al. 2016	---
Camellia sinensis	16	---	No	Yu et al. 2020	---
Capsicum annuum	33	---	No	Wu et al. 2016	---
Capsicum annuum	33	Segmental duplication	Ka/Ks ratios	Kang et al. 2016	Purifying selection
Carica papaya	20	---	No	Shangguan et al. 2020	---
Chrysantemum morifolium	20*	---	No	Song et al. 2016	---
Cicer arietinum	37	Segmental duplication	Yes	Nasim et al. 2016	Multiple sites under positive selection; Ka/Ks ranging from 0.123 to 1.026
Citrullus lanatus	36	Segmental duplication	No	Zhou et al. 2020	---
Citrus sinensis	24	---	No	Guaberto et al. 2019	---
Citrus sinensis	24	---	No	Shangguan et al. 2020	---
Cleistogenes songorica	50	Segmental duplication	Ka/Ks ratios	Wang et al. 2021	Purifying selection
Cucumis sativus	36	Segmental duplication	No	Wen et al. 2016	---
Daucus carota	46	---	No	Huang et al. 2015	---
Durio zibethinus	24	---	No	Khaksar et al. 2019	---
Eleusine coracana	48	---	No	Gupta et al. 2018	---
Eragrostis tef	33	---	No	Mulat et al. 2020	---
Eugenia uniflora	28	---	No	Waschburger et al. 2022	---
Fragaria vesca	23	---	No	Shangguan et al. 2020	---
Glycine max	76	---	No	Wang et al. 2019	---
Gossypium arboreum	58	---	No	Chattha et al. 2020	---
Gossypium	55	Segmental duplication	Ka/Ks ratios	Li et al. 2020	Purifying selection

Species	DOFs members	Main source of diversification	Selection analysis	Reference	Predominant selective force
arboreum					
Gossypium barbadense	110	---	No	Chattha et al. 2020	---
Gossypium barbadense	116	Segmental duplication	Ka/Ks ratios	Li et al. 2020	Purifying selection
Gossypium hirsutum	89	---	No	Chattha et al. 2020	---
Gossypium hirsutum	115	Segmental duplication	Ka/Ks ratios	Li et al. 2020	Purifying selection
Gossypium hirsutum	114	Segmental duplication	No	Li et al. 2018	---
Gossypium raimondii	55	---	No	Chattha et al. 2020	---
Gossypium raimondii	56	Segmental duplication	Ka/Ks ratios	Li et al. 2020	Purifying selection
Helianthus annuus	46	---	No	Wang et al. 2019	---
Hevea brasiliensis	46	Segmental duplication	Ka/Ks ratios	Zou and Yang 2019	Purifying selection
Jatropha curcas	24	Segmental duplication	Ka/Ks ratios	Wang et al. 2018	Positive selection
Jatropha curcas	25	Tandem duplication	No	Zou and Zhang 2019	---
Juglans regia	39	---	No	Khan et al. 2021	---
Kobresia littledalei	25	Tandem duplication	---	Yu et al. 2021	Not calculated for duplicate pairs of this species
Malus domestica	59	---	No	Shangguan et al. 2020	---
Malus domestica	60	Segmental duplication	Ka/Ks ratios	Zhang et al. 2018	Purifying selection
Malus domestica	54	---	No	Yang et al. 2018	---
Manihot esculenta	45	Segmental duplication	Ka/Ks ratios	Zou et al. 2019	Purifying selection
Medicago sativa	40	---	Ka/Ks ratios	Cao et al. 2020	Purifying selection
Medicago truncatula	42	Tandem and Segmental duplication	No	Shu et al. 2015	---
Musa	25	---	No	Feng et al. 2016	---

Species	DOFs members	Main source of diversification	Selection analysis	Reference	Predominant selective force
acuminata					
Musa acuminata	74	Segmental duplication	No	Dong et al. 2016	---
Musa acuminata	73	Segmental duplication	Ka/Ks ratios	Shangguan et al. 2020	Purifying selection
Nelumbo nucifera	29	Segmental duplications	---	Cao et al. 2022	Purifying selection
Oryza barthii	19		Ka/Ks ratios	Tabassum et al. 2022	
Oryza brachyantha	09		Ka/Ks ratios	Tabassum et al. 2022	
Oryza glaberrima	23		Ka/Ks ratios	Tabassum et al. 2022	
Oryza glumipatula	28		Ka/Ks ratios	Tabassum et al. 2022	Substitution rates from duplicated gene pairs calculated also between different rice species. 85% of the duplicated pairs were under purifying selection and 12% were under positive selection
Oryza meridionalis	25		Ka/Ks ratios	Tabassum et al. 2022	
Oryza nivara	30	Segmental duplications	Ka/Ks ratios	Tabassum et al. 2022	
Oryza punctata	25		Ka/Ks ratios	Tabassum et al. 2022	
Oryza rufipogon	27		Ka/Ks ratios	Tabassum et al. 2022	
Oryza sativa indica	27		Ka/Ks ratios	Tabassum et al. 2022	
Oryza sativa japonica	25		Ka/Ks ratios		
Oryza sativa indica ZS97	28	---	---	Tabassum et al. 2022	---
Oryza sativa indica 93-11	27	---	---	Tabassum et al. 2022	---
Oryza sativa indica R498	27	---	---	Tabassum et al. 2022	---
Oryza sativa indica MH63	23	---	---	Tabassum et al. 2022	---
Oryza sativa	30	---	No	Lijavetzki et al. 2003	---
Oryza sativa	25	---	No	Wang et al. 2019	---

Species	DOFs members	Main source of diversification	Selection analysis	Reference	Predominant selective force
Oryza sativa	30	Segmental duplication	Ka/Ks ratios	Shangguan et al. 2020	Purifying selection
Oryza sativa	30	---	No	Liu et al. 2021	---
Oryza sativa	30	---	No	Khan et al. 2021	---
Petunia inflata	35	---	No	Yue et al. 2021	---
Phaseolus vulgaris	36	Segmental duplication	Ka/Ks ratios	Ito et al. 2017	Purifying selection
Phyllostachys heterocycla	26	---	No	Wang et al. 2016	---
Physcomitrella patens	19	---	No	Shigyo et al. 2007	---
Populus trichocarpa	45	Segmental duplication	Ka/Ks ratios	Shangguan et al. 2020	Purifying selection
Populus trichocarpa	41	Segmental duplication	No	Wang et al. 2017	---
Populus trichocarpa	44	Probably homologous recombination	Selecton server	Wang et al. 2022	Under a substantial amount of purifying selection
Prunus persica	25	---	No	Shangguan et al. 2020	---
Prunus persica	25	Probably segmental duplication	No	Chen et al. 2016	---
Pyrus bretschneideri	45	---	No	Liu et al. 2019	---
Pyrus bretschneideri	46	---	No	Shangguan et al. 2020	---
Ricinus communis	24	---	No	Zou and Zhang 2019	---
Ricinus communis	21	---	No	Jin et al. 2014	---
Rosa chinensis	24	Segmental duplication	Ka/Ks ratios	Nan et al. 2021	Purifying selection
Saccharum spontaneum	29/89	Segmental duplication	Ka/Ks ratios	Cai et al. 2020	Purifying selection
Setaria italica	35	---	No	Zhang et al. 2017	---
Setaria italica	26	---	No	Wang et al. 2019	---
Setaria viridis	35	---	No	Wang et al. 2019	---
Solanum lycopersicum	31	---	No	Wang et al. 2019	---

**Species**	**DOFs members**	**Main source of diversification**	**Selection analysis**	**Reference**	**Predominant selective force**
Solanum lycopersicum	34	Segmental duplication	Ka/Ks ratios	Cai et al. 2013	Purifying selection
Solanum lycopersicum	32	Segmental duplication	Ka/Ks ratios	Shangguan et al. 2020	Purifying selection
Solanum melongena	29	---	No	Wei et al. 2018	---
Solanum tuberosum	35	Probably tandem duplication	No	Venkatesh and Park 2015	---
Sorghum bicolor	30	---	No	Wang et al. 2019	---
Sorghum bicolor	28	---	No	Kushwaha et al. 2011	---
Sorghum bicolor	30	---	No	Xiao et al. 2022	---
Spinacia oleracea	22	---	Ka/Ks ratios	Yu et al. 2021	Purifying selection
Triticum aestivum	31	---	No	Shaw et al. 2009	---
Triticum aestivum	96	Tandem duplication	Yes	Liu et al. 2020	Five codons under Positive selection
Triticum aestivum	108	Segmental duplication	Ka/Ks ratios	Fang et al. 2020	Purifying selection
Vaccinium corymbosum	51	whole-genome duplication	Ka/Ks ratios	Li et al. 2022	91.67% gene pairs under purifying selection and two genes pairs under positive selection
Vitis vinifera	25	Segmental duplication	Ka/Ks ratios	Shangguan et al. 2020	Purifying selection
Vitis vinifera	25	Segmental duplication	No	Wang et al. 2021	---
Vitis vinifera	25	---	No	da Silva et al. 2016	---
Zea mays	48	---	No	Wang et al. 2019	---
Zea mays subsp. mays	46	Segmental duplication	Yes	Chen and Cao 2015	Multiple sites under positive selection; Ka/Ks ranging from 0.214 to 0.827
