# Supplementary material for: DOF gene family expansion and diversification
Source: Genet Mol Biol. 2024 Feb 5;46(3 Suppl 1):e20230109. doi: 10.1590/1678-4685-GMB-2023-0109 (PMC10842470; doi:10.1590/1678-4685-GMB-2023-0109)
Supplement: Table S3 - [file 1415-4757-GMB-46-03-s1-e20230109-s5.pdf]

Genetics and Molecular Biology
https://doi.org/10.1590/1678-4685-GMB-2023-0109

Supplementary Material to "DOF gene family expansion and diversification"

Table S3 - Comparison of DOF homologous groups among studies.

Sequences	DOF	Alias	Yanagisawa 2002	Lijavetzky et al., 2003	Moreno-Risueno et al., 2007	Waschburger et al., 2023
AT1G29160	1.5	COG1	II	D1	A	G02
AT2G34140	2.3	cDOF4	II	D1	A	G02
AT1G69570	1.10	cDOF5	II	D1	A	G02.1
AT1G26790	1.3	cDOF6	II	D1	A	G02.1
AT3G47500	3.3	cDOF3	II	D1	A	G02.1
AT5G39660	5.2	cDOF2	II	D1	A	G02.1
AT5G62430	5.5	cDOF1	II	D1	A	G02.1
AT1G07640	1.1	OBP2	V	A	G	G03
AT2G28810	2.2		V	B	G	G03
AT2G37590	2.4	PEAR1	V	B	G	G03
AT3G55370	3.6	OBP3	V	B	G	G03
AT5G02460	5.1	PEAR2	V	B	G	G03
AT1G28310	1.4		IV	B	E	G04
AT1G47655	1.6		IV	D2	D	G06
AT3G50410	3.4	OBP1	IV	D2	D	G06
AT5G66940	5.8	DOF5.8	IV	D2	D	G06
AT5G60850	5.4	OBP4	VI	A	B	G07
AT1G21340	1.2		III	C2.2	F	G08
AT3G52440	3.5		III	C2.2	F	G08
AT5G62940	5.6	HCA2	VII	C1	F	G09
AT3G45610	3.2	DOF6	III	C1	F	G10
AT1G64620	1.8	VDOF2	III	C2.1	F	G10
AT3G61850	3.7	DAG1	III	C2.1	F	G10
AT4G00940	4.1	ITD1	III	C2.1	F	G10
AT4G24060	4.6	VDOF1	III	C2.1	F	G10
AT1G51700	1.7	ADOF1	IV	A	D	Ungrouped
AT3G21270	3.1	ADOF2	IV	A	D	Ungrouped
AT5G65590	5.7	SCAP1	IV	A	D	Ungrouped
AT4G38000	4.7		IV	B	D	Ungrouped
AT4G21030	4.2	DOF4.2	I	C3	F	Ungrouped
AT4G21040	4.3		I	C3	F	Ungrouped
AT4G21050	4.4		I	C3	F	Ungrouped
AT4G21080	4.5		I	C3	F	Ungrouped
AT2G28510	2.1	DOF2.1	III	C1	F	Ungrouped
AT5G60200	5.3	TMO6	III	C1	F	Ungrouped
AT2G46590	2.5	DAG2	III	C2.1	F	Ungrouped
