# Supplementary material for: DOF gene family expansion and diversification
Source: Genet Mol Biol. 2024 Feb 5;46(3 Suppl 1):e20230109. doi: 10.1590/1678-4685-GMB-2023-0109 (PMC10842470; doi:10.1590/1678-4685-GMB-2023-0109)
Supplement: Table S4 - [file 1415-4757-GMB-46-03-s1-e20230109-s6.pdf]

Genetics and Molecular Biology
https://doi.org/10.1590/1678-4685-GMB-2023-0109

Supplementary Material to “DOF gene family expansion and diversification”

Table S4 - Phylogenetic groups sequence numbers.

Clade I	Code I	Clade II	Code II	Order	Family	Species	Total Seqs	Filtered	Phylogenetic Groups: G1	G2	G2.1	G3	G4	G5	G6	G7	G8	G9	G10	Ungrouped
ALGAE	AL	-	-	CHARALES	Characeae	*Chara braunii*	1	1	0	1	0	0	0	0	0	0	0	0	0	0
ALGAE	AL	-	-	CHLAMYDOMONADALES	Chlamydomonadaceae	*Chlamydomonas reinhardtii*	1	1	0	1	0	0	0	0	0	0	0	0	0	0
ALGAE	AL	-	-	CHLAMYDOMONADALES	Volvocaceae	*Volvox carteri*	1	1	0	1	0	0	0	0	0	0	0	0	0	0
ALGAE	AL	-	-	KLEBSORMIDIALES	Klebsormidiaceae	*Klebsormidium nitens*	1	1	0	1	0	0	0	0	0	0	0	0	0	0
ALGAE	AL	-	-	MAMIELLALES	Bathycoccaceae	*Ostreococcus lucimarinus*	2	2	0	1	0	0	0	0	0	0	0	0	0	1
ALGAE	AL	-	-	MAMIELLALES	Mamiellaceae	*Micromonas pusilla*	1	1	0	1	0	0	0	0	0	0	0	0	0	0
ALGAE	AL	-	-	SPHAEROPLEALES	Chromochloridaceae	*Chromochloris zofingiensis*	1	1	0	1	0	0	0	0	0	0	0	0	0	0
ALGAE	AL	-	-	TREBOUXIALES	Botryococcaceae	*Botryococcus braunii*	1	1	0	1	0	0	0	0	0	0	0	0	0	0
ANCIENT ANGIOSPERMS	AA	-	-	AMBORELLALES	Amborellaceae	*Amborella trichopoda*	18	17	0	1	2	1	1	0	1	1	1	0	1	8
ANCIENT ANGIOSPERMS	AA	-	-	LAURALES	Lauraceae	*Cinnamomum kanehirae*	29	28	1	1	5	3	1	0	5	2	0	3	2	5
ANCIENT ANGIOSPERMS	AA	-	-	NYMPHAEALES	Nymphaceae	*Nymphaea colorata*	27	27	2	1	5	2	2	0	1	1	1	1	2	9
BRYOPHYES	BR	-	-	DICRANALES	Ditrichaceae	*Ceratodon purpureus*	15	13	0	10	0	0	0	0	0	0	0	0	0	3
BRYOPHYES	BR	-	-	FUNARIALES	Funariaceae	*Physcomitrella patens*	27	26	0	17	0	0	0	0	0	0	0	0	0	7
BRYOPHYES	BR	-	-	MARCHANTIALES	Marchantiaceae	*Marchantia polymorpha*	2	2	0	2	0	0	0	0	0	0	0	0	0	0
BRYOPHYES	BR	-	-	SPHAGNALES	Sphagnaceae	*Sphagnum fallax*	24	24	0	24	0	0	0	0	0	0	0	0	0	0
BRYOPHYES	BR	-	-	SPHAGNALES	Sphagnaceae	*Sphagnum magellanicum*	24	24	0	24	0	0	0	0	0	0	0	0	0	0
EUDICOTS	EU	ANCIENT EUDICOTS	ANCEU	RANUNCULALES	Papaveraceae	*Papaver somniferum*	35	34	1	1	4	2	4	2	3	1	3	2	1	10
EUDICOTS	EU	ANCIENT EUDICOTS	ANCEU	RANUNCULALES	Ranunculaceae	*Aquilegia coerulea*	29	29	2	6	1	2	1	0	2	1	2	2	1	9
EUDICOTS	EU	ASTERID I	AST1	GENTIANALES	Apocynaceae	*Calotropis gigantea*	24	23	1	1	3	3	1	1	2	1	1	1	4	4
EUDICOTS	EU	ASTERID I	AST1	GENTIANALES	Rubiaceae	*Coffea canephora*	28	23	1	1	3	3	1	1	2	1	2	1	4	3
EUDICOTS	EU	ASTERID I	AST1	LAMIALES	Bignoiaceae	*Handroanthus impetiginosus*	36	35	0	2	4	2	3	2	4	2	1	2	4	9
EUDICOTS	EU	ASTERID I	AST1	LAMIALES	Lamiaceae	*Salvia miltiorrhiza*	26	20	1	2	3	2	1	1	1	0	3	0	3	3
EUDICOTS	EU	ASTERID I	AST1	LAMIALES	Oleaceae	*Olea europaea*	53	52	1	2	7	5	2	2	2	2	4	3	8	14

Clade I	Code I	Clade II	Code II	Order	Family	Species	Total Seqs	Filtered												
EUDICOTS	EU	ASTERID I	AST1	LAMIALES	Orobanchaceae	*Lindenbergia philippensis*	33	32	1	2	5	2	2	1	2	0	2	1	4	10
EUDICOTS	EU	ASTERID I	AST1	LAMIALES	Pedaliaceae	*Sesamum indicum*	31	31	1	1	5	2	2	2	3	2	2	1	2	8
EUDICOTS	EU	ASTERID I	AST1	LAMIALES	Phrymaceae	*Mimulus guttatus*	35	35	1	2	4	2	2	1	3	1	2	1	3	13
EUDICOTS	EU	ASTERID I	AST1	SOLANALES	Convolvulaceae	*Ipomoea triloba*	47	36	1	2	6	6	1	2	3	2	2	1	4	6
EUDICOTS	EU	ASTERID I	AST1	SOLANALES	Solanaceae	*Solanum lycopersicum*	31	31	1	1	4	5	1	1	3	1	1	2	5	6
EUDICOTS	EU	ASTERID II	AST2	APIALES	Apiaceae	*Daucus carota*	42	42	1	2	6	7	3	1	3	1	2	1	3	12
EUDICOTS	EU	ASTERID II	AST2	APIALES	Araliaceae	*Panax notoginseng*	13	13	1	1	1	3	2	0	1	1	0	0	0	3
EUDICOTS	EU	ASTERID II	AST2	ASTERALES	Asteraceae	*Lactuca sativa*	38	38	2	1	5	4	1	2	2	2	2	2	5	10
EUDICOTS	EU	ASTERIDS	AST	CORNALES	Hydrangeaceae	*Hydrangea quercifolia*	42	40	2	3	7	4	2	1	3	2	3	1	5	7
EUDICOTS	EU	ASTERIDS	AST	CORNALES	Nyssaceae	*Camptotheca acuminata*	54	40	2	2	7	5	1	2	4	2	2	2	5	6
EUDICOTS	EU	ASTERIDS	AST	ERICALES	Actinidiaceae	*Actinidia chinensis*	53	53	2	2	10	8	2	2	8	2	2	2	5	8
EUDICOTS	EU	ASTERIDS	AST	ERICALES	Ericaceae	*Rhododendron delavayi*	30	30	1	1	5	4	1	1	5	1	2	1	5	3
EUDICOTS	EU	ASTERIDS	AST	ERICALES	Theaceae	*Camellia sinensis*	19	19	0	1	4	1	2	1	0	1	1	2	3	3
EUDICOTS	EU	ROSID	ROS	SAXIFRAGALES	Crassulaceae	*Kalanchoe fedtschenkoi*	41	39	1	2	6	5	3	0	1	2	2	1	5	11
EUDICOTS	EU	ROSID	ROS	VITALES	Vitaceae	*Vitis vinifera L.*	25	25	1	1	4	3	1	1	3	1	2	1	3	4
EUDICOTS	EU	ROSID I	ROS1	CUCURBITALES	Cucurbitaceae	*Cucumis sativus*	41	36	1	2	5	6	2	1	3	1	2	1	5	7
EUDICOTS	EU	ROSID I	ROS1	FABALES	Fabaceae	*Medicago truncatula*	42	38	1	1	12	5	2	2	1	2	2	1	4	5
EUDICOTS	EU	ROSID I	ROS1	FAGALES	Betulaceae	*Betula platyphylla*	26	25	1	1	5	3	1	1	3	1	2	0	4	3
EUDICOTS	EU	ROSID I	ROS1	FAGALES	Fagaceae	*Quercus lobata*	27	27	1	1	5	4	1	1	2	1	2	1	3	5
EUDICOTS	EU	ROSID I	ROS1	FAGALES	Juglandaceae	*Juglans regia*	54	52	2	2	9	8	2	2	7	2	4	1	6	7
EUDICOTS	EU	ROSID I	ROS1	MALPHIGIALES	Salicaceae	*Populus trichocarpa*	42	42	2	2	6	5	2	2	4	2	4	2	6	5
EUDICOTS	EU	ROSID I	ROS1	MALPIGHIALES	Euphorbiaceae	*Ricinus communis*	23	21	1	1	3	1	1	1	2	1	2	1	3	4
EUDICOTS	EU	ROSID I	ROS1	MALPIGHIALES	Linaceae	*Linum usitatissimum*	43	41	0	2	6	6	0	2	2	2	4	2	7	8
EUDICOTS	EU	ROSID I	ROS1	OXALIDALES	Cephalotaceae	*Cephalotus follicularis*	21	21	1	1	3	3	1	1	3	1	1	1	2	3
EUDICOTS	EU	ROSID I	ROS1	ROSALES	Cannabaceae	*Cannabis sativa*	23	18	0	1	5	3	0	0	2	1	0	1	2	3
EUDICOTS	EU	ROSID I	ROS1	ROSALES	Moraceae	*Ficus carica*	25	24	1	1	4	3	1	1	2	1	2	1	3	4
EUDICOTS	EU	ROSID I	ROS1	ROSALES	Rhamnaceae	*Ziziphus jujuba (Chinese jujube)*	32	31	2	1	6	4	2	1	2	1	2	1	4	5
EUDICOTS	EU	ROSID I	ROS1	ROSALES	Rosaceae	*Fragaria vesca*	25	24	1	1	3	3	1	1	2	1	2	1	3	5
EUDICOTS	EU	ROSID II	ROS2	BRASSICALES	Brassicaceae	*Arabidopsis thaliana*	36	36	0	2	5	5	1	0	4	1	2	1	5	10
EUDICOTS	EU	ROSID II	ROS2	BRASSICALES	Caricaceae	*Carica papaya*	20	19	1	0	3	2	1	1	1	1	2	1	2	4
EUDICOTS	EU	ROSID II	ROS2	BRASSICALES	Cleomaceae	*Cleome violacea*	26	26	0	1	4	4	1	0	2	1	2	1	4	6
EUDICOTS	EU	ROSID II	ROS2	CARYOPHILLALES	Amaranthaceae	*Beta vulgaris*	20	19	1	1	4	2	1	1	0	1	3	1	2	2
EUDICOTS	EU	ROSID II	ROS2	CARYOPHILLALES	Polygonaceae	*Fagopyrum tataricum*	36	35	1	2	5	4	1	1	3	2	4	2	4	6
EUDICOTS	EU	ROSID II	ROS2	CARYOPHILLALES	Portulacaceae	*Portulaca amilis*	30	30	2	1	5	1	2	2	2	2	2	1	4	6
EUDICOTS	EU	ROSID II	ROS2	MALVALES	Malvaceae	*Theobroma cacao*	25	25	1	1	5	3	1	1	3	1	2	1	3	3
EUDICOTS	EU	ROSID II	ROS2	MYRTALES	Lythraceae	*Punica granatum*	29	28	0	2	5	4	1	1	3	2	2	1	3	4
EUDICOTS	EU	ROSID II	ROS2	MYRTALES	Myrtaceae	*Corymbia citriodora*	28	28	1	1	5	3	1	1	4	1	2	1	4	4
EUDICOTS	EU	ROSID II	ROS2	SAPINDALES	Anacardiaceae	*Anacardium occidentale*	51	50	2	1	9	6	2	2	3	3	4	1	8	9
EUDICOTS	EU	ROSID II	ROS2	SAPINDALES	Anacardiaceae	*Pistacia vera*	27	26	1	1	5	3	1	1	3	1	1	2	3	4
EUDICOTS	EU	ROSID II	ROS2	SAPINDALES	Meliaceae	*Azadirachta indica*	34	29	1	2	6	3	2	1	1	1	3	1	4	4
EUDICOTS	EU	ROSID II	ROS2	SAPINDALES	Rutaceae	*Citrus sinensis*	24	24	1	1	4	3	1	1	2	1	2	1	3	4
EUDICOTS	EU	ROSID II	ROS2	SAPINDALES	Sapindaceae	*Dimocarpus longan*	23	23	1	1	4	3	1	1	2	1	2	1	3	3
GYMNOSPERMS	GM	-	-	GINKGOALES	Ginkgoaceae	*Ginkgo biloba*	14	14	1	3	1	0	2	0	0	1	1	0	0	5
GYMNOSPERMS	GM	-	-	PINALES	Cupressaceae	*Thuja plicata*	21	18	0	4	5	0	0	0	0	2	0	0	0	7

Clade I	Code I	Clade II	Code II	Order	Family	Species	Total Seqs	Filtered												
GYMNOSPERMS	GM	-	-	PINALES	Pinaceae	*Picea abies*	23	22	1	2	9	0	1	0	0	1	0	0	0	8
~~GYMNOSPERMS~~	~~GM~~	~~-~~	~~-~~	~~PINALES~~	~~Pinaceae~~	~~*Picea glauca*~~	~~0~~	~~0~~	~~0~~	~~0~~	~~0~~	~~0~~	~~0~~	~~0~~	~~0~~	~~0~~	~~0~~	~~0~~	~~0~~	~~0~~
GYMNOSPERMS	GM	-	-	PINALES	Pinaceae	*Pinus taeda*	8	8	0	0	2	0	2	0	0	2	0	0	0	2
MONOCOTS	MN	COMELINIDS	COM	ARECALES	Arecaceae	*Phoenix dactylifera*	19	16	2	0	5	0	0	0	0	0	2	2	2	3
MONOCOTS	MN	COMELINIDS	COM	POALES	Bromeliaceae	*Ananas comosus L.*	26	23	0	1	4	3	1	0	0	0	1	1	2	10
MONOCOTS	MN	COMELINIDS	COM	POALES	Poaceae	*Oryza sativa*	30	30	0	2	5	5	0	0	1	2	3	1	2	9
MONOCOTS	MN	COMELINIDS	COM	ZINGIBERALES	Musaceae	*Musa acuminata L.*	74	73	4	4	11	12	4	0	4	3	3	2	10	16
MONOCOTS	MN	MONOCOTS	MON	ALISMATALES	Araceae	*Spirodela polyrhiza*	26	25	2	0	5	4	2	0	1	0	1	1	3	6
MONOCOTS	MN	MONOCOTS	MON	ALISMATALES	Zosteraceae	*Zostera marina*	28	28	0	2	3	4	2	0	0	1	2	1	2	11
MONOCOTS	MN	MONOCOTS	MON	ASPARAGALES	Asparagaceae	*Asparagus officinalis*	4	2	0	0	1	0	0	0	0	0	0	0	1	0
MONOCOTS	MN	MONOCOTS	MON	ASPARAGALES	Orchidaceae	*Cypripedium arietinum*	34	32	1	1	9	5	2	2	1	2	1	1	1	6
MONOCOTS	MN	MONOCOTS	MON	DIOSCOREALES	Dioscoreaceae	*Dioscorea alata*	35	35	2	3	6	4	1	0	1	3	1	2	2	10
MONOCOTS	MN	MONOCOTS	MON	PANDANALES	Velloziaceae	*Xerophyta viscosa*	36	36	0	0	5	8	1	0	0	0	0	1	8	13
SPORULATING TRACHEOPHYTES	ST	-	-	POLYPODIALES	Pteridaceae	*Ceratopteris richardii*	26	25	0	15	0	0	0	0	0	0	0	0	0	10
SPORULATING TRACHEOPHYTES	ST	-	-	SALVINIALES	Salviniaceae	*Salvinia cucullata*	14	13	0	8	1	0	1	0	0	0	0	0	0	3
SPORULATING TRACHEOPHYTES	ST	-	-	SELAGINELLALES	Selaginellanaceae	*Selaginella moellendorffii*	37	34	0	4	1	0	0	4	0	0	0	0	0	25
							2231	2122	68	210	330	231	95	63	143	86	122	76	224	472
