# Supplementary material for: DOF gene family expansion and diversification
Source: Genet Mol Biol. 2024 Feb 5;46(3 Suppl 1):e20230109. doi: 10.1590/1678-4685-GMB-2023-0109 (PMC10842470; doi:10.1590/1678-4685-GMB-2023-0109)
Supplement: Material S1 - [file 1415-4757-GMB-46-03-s1-e20230109-s7.pdf]

Genetics and Molecular Biology
https://doi.org/10.1590/1678-4685-GMB-2023-0109

Supplementary Material to "DOF gene family expansion and diversification"

Material S1 - Filtered sequences.

Removed Sequences:

Ricinus communis:
EU_ROS1_Ricom_30025_t000009

Hyque:
EU_AST_Hyque_Hyque_14G108500

Cyari:
MN_MON_Cyari_Ca_04005
MN_MON_Cyari_Ca_03849

Cocan:
EU_AST1_Cocan_GSCOC_T00004809001

Capap:
EU_ROS2_Capap_evm_model_supercontig_96_59

Cikan:
AA_Cikan_CKAN_00944100

Azind:
EU_ROS2_Azind_Neem_14014
EU_ROS2_Azind_Neem_22176
EU_ROS2_Azind_Neem_40102
EU_ROS2_Azind_Neem_5458

Piabi:
GM_Piabi_MA_248613g0010
