# Supplementary material for: DOF gene family expansion and diversification
Source: Genet Mol Biol. 2024 Feb 5;46(3 Suppl 1):e20230109. doi: 10.1590/1678-4685-GMB-2023-0109 (PMC10842470; doi:10.1590/1678-4685-GMB-2023-0109)
Supplement: Material S2 - [file 1415-4757-GMB-46-03-s1-e20230109-s8.pdf]

Genetics and Molecular Biology
https://doi.org/10.1590/1678-4685-GMB-2023-0109

Supplementary Material to “DOF gene family expansion and diversification”

Material S2 - Materials and methods.

Literature survey

NIH’s PubMed (2023, pubmed.ncbi.nlm.nih.gov) and Thomson Scientific’s Web of Science (2023, apps.webofknowledge.com) were selected as the on-line libraries for published scientific research articles retrieval. Full text articles mutually containing the keywords “Transcription Factor” and “DOF” were downloaded from these libraries on February 18th, 2023, followed by removal of any that didn’t have the plant-specific DOF transcription factor family as the main focus of research. The two generated databases were then merged, and duplicated articles were excluded. All articles were manually annotated, and the figures constructed using the resulting spreadsheet’s information. All illustrations were made using Adobe Illustrator CC (2022, adobe.com).

Sequence retrieval

A list of every plant species available across the genome databases Phytozome v13 (2022, phytozome-next.jgi.doe.gov), Ensembl Plants (2022, plants.ensembl.org), and KEGG Genomes (2022, genome.jp/kegg/genome), as well as the TF database iTak (2022, itak.feilab.net), was assembled and used to identify representative organisms of the many botanical families. One species of every family was selected for further sequence retrieval and future analyses (Table S3). The order Phytozome, Ensembl Plants, iTak, and KEGG Genomes was followed to determine from which database redundant species sequences (such as the sequences from *Arabidopsis thaliana*) were going to be recovered. Klebsormidium nitens and Pinus species were later added (from PlantMorphogenesis, 2022, http://www.plantmorphogenesis.bio.titech.ac.jp/~algae_genome_project/klebsormidium and PlantGenie, 2022, plantgenie.org respectively). A HMMER search using the DOF HMM profile (PF02701) was performed to retrieve all available DOF genes from the primary transcript protein fasta files (Eddy, 2011).

Alignment and phylogeny reconstruction

The DOF domain logo was generated using WebLogo (Crooks *et al.*, 2004). All alignments were conducted using MAFFT by the L-INS-i algorithm (Katoh *et al.*, 2019). Sequences lacking the DOF domain, totally or partially, were excluded from further analyses (Material S1). IQTree v2.2.0 (Minh *et al.*, 2020) was used to estimate the DOF phylogenetic tree by the maximum-likelihood (ML) method with 10,000 UFBootstrap (Hoang *et al.*, 2018). Three independent runs were done and the resulting tree with the highest ML score was selected. The best substitution model for amino acids was determined using ModelFinder included in IQTree (Kalyaanamoorthy *et al.*, 2017). Phylogeny visualization was performed using the FigTree software (2022, tree.bio.ed.ac.uk/software/figtree).

References

Crooks GE, Hon G, Chandonia J-M and Brenner SE (2004) WebLogo: A sequence logo generator. Genome Res 14:1188-1190.

Eddy SR (2011) Accelerated profile HMM searches. PLoS Comput Biol 7:e1002195.

Hoang DT, Chernomor O, Von Haeseler A, Minh BQ and Vinh LS (2018) UFBoot2: Improving the ultrafast bootstrap approximation. Mol Biol Evol 35:518–522.

Kalyaanamoorthy S, Minh BQ, Wong TKF, von Haeseler A and Jermiin LS (2017) ModelFinder: Fast model selection for accurate phylogenetic estimates. Nat Methods 14:587-589.

Katoh K, Rozewicki J and Yamada KD (2019) MAFFT online service: Multiple sequence alignment, interactive sequence choice and visualization. Brief Bioinform 20:1160-1166.

Minh BQ, Schmidt HA, Chernomor O, Schrempf D, Woodhams MD, von Haeseler A and Lanfear R (2020) IQ-TREE 2: New models and efficient methods for phylogenetic inference in the genomic era. Mol Biol Evol 37:1530-1534.

Internet Resources

Pubmed (2023) Pubmed, https://pubmed.ncbi.nlm.nih.gov/ (accessed 18 February 2023)

Web of Science (2023) Clarivate, https://access.clarivate.com/login?app=wos&alternative=true&shibShireURL=https:%2F%2Fwww.webofknowledge.com%2F%3Fauth%3DShibboleth&shibReturnURL=https:%2F%2Fwww.webofknowledge.com%2F&roaming=true (accessed 18 February 2023)

Phytozome (2022) The Plant Genomics Resource, https://phytozome-next.jgi.doe.gov/ (accessed 28 November 2022)

Ensembl Plants (2022) EsemblPlants, https://plants.ensembl.org/index.html (accessed 28 November 2022)

KEGG Genomes (2022) https://www.genome.jp/kegg/genome/ (accessed 28 November 2022)

iTak (2022) iTak, http://itak.feilab.net/cgi-bin/itak/index.cgi (accessed 28 November 2022)

PlantMorphogenesis (2022) http://www.plantmorphogenesis.bio.titech.ac.jp/~algae_genome_project/klebsormidium (accessed 28 November 2022)

PlantGenie (2022) PlantGenie.org, https://plantgenie.org/ (accessed 28 November 2022)

WebLogo (2023) WebLogo, https://weblogo.berkeley.edu/logo.cgi (accessed 4 February 2023)

MAFFT (2023) MAFFT version 7, https://mafft.cbrc.jp/alignment/software/ (accessed 28 November 2022)

IQTree (2022) Iq-Tree, http://www.iqtree.org/ (accessed 29 November 2022)

FigTree (2022) Molecular Evolution, Phylogenetics and Epidemiology, http://tree.bio.ed.ac.uk/software/figtree/ (accessed 1 December 2022)

Adobe Illustrator (2022) Adobe, https://www.adobe.com/ (accessed 23 March 2022)
